# Efficiency of Vanilla, Patchouli and Ylang Ylang Essential Oils Stabilized by Iron Oxide@C_14_ Nanostructures against Bacterial Adherence and Biofilms Formed by *Staphylococcus aureus* and *Klebsiella pneumoniae* Clinical Strains

**DOI:** 10.3390/molecules191117943

**Published:** 2014-11-04

**Authors:** Maxim Bilcu, Alexandru Mihai Grumezescu, Alexandra Elena Oprea, Roxana Cristina Popescu, George Dan Mogoșanu, Radu Hristu, George A. Stanciu, Dan Florin Mihailescu, Veronica Lazar, Eugenia Bezirtzoglou, Mariana Carmen Chifiriuc

**Affiliations:** 1 Microbiology Department, Faculty of Biology, Research Institute of the University of Bucharest, University of Bucharest, Aleea Portocalelor No. 1–3, 060101 Bucharest, Romania; E-Mails: spartacus2007@yahoo.com (M.B.); d.f.mihailescu@gmail.com (D.F.M.); veronica.lazar2009@gmail.com (V.L.); carmen_balotescu@yahoo.com (M.C.C.); 2 Department of Science and Engineering of Oxide Materials and Nanomaterials, Faculty of Applied Chemistry and Materials Science, University Politehnica of Bucharest, Polizu Street No. 1–7, 011061 Bucharest, Romania; E-Mails: grumezescu@yahoo.com (A.M.G.); elena_oprea_93@yahoo.co.uk (A.E.O.); roxpopescu@yahoo.co.uk (R.C.P.); 3 Department of Pharmacognosy & Phytotherapy, Faculty of Pharmacy, University of Medicine and Pharmacy of Craiova, 2 Petru Rareş Street, 200349 Craiova, Romania; E-Mail: george.mogosanu@umfcv.ro; 4 Center for Microscopy-Microanalysis and Information Processing, University Politehnica of Bucharest, 313 Splaiul Independentei, 060042 Bucharest, Romania; E-Mails: hristu_radu@yahoo.com (R.H.); stanciu@physics.pub.ro (G.A.S.); 5 Laboratory of Microbiology, Biotechnology and Hygiene, Department of Food Science and Technology, Faculty of Agricultural Development, Democritus University of Thrace, 68200 Orestiada, Greece

**Keywords:** magnetite, essential oil, adherence, anti-biofilm

## Abstract

Biofilms formed by bacterial cells are associated with drastically enhanced resistance against most antimicrobial agents, contributing to the persistence and chronicization of the microbial infections and to therapy failure. The purpose of this study was to combine the unique properties of magnetic nanoparticles with the antimicrobial activity of three essential oils to obtain novel nanobiosystems that could be used as coatings for catheter pieces with an improved resistance to *Staphylococcus aureus* and *Klebsiella pneumoniae* clinical strains adherence and biofilm development. The essential oils of ylang ylang, patchouli and vanilla were stabilized by the interaction with iron oxide@C_14_ nanoparticles to be further used as coating agents for medical surfaces. Iron oxide@C_14_ was prepared by co-precipitation of Fe^+2^ and Fe^+3^ and myristic acid (C_14_) in basic medium. Vanilla essential oil loaded nanoparticles pelliculised on the catheter samples surface strongly inhibited both the initial adherence of *S. aureus* cells (quantified at 24 h) and the development of the mature biofilm quantified at 48 h. Patchouli and ylang-ylang essential oils inhibited mostly the initial adherence phase of *S. aureus* biofilm development. In the case of *K. pneumoniae*, all tested nanosystems exhibited similar efficiency, being active mostly against the adherence *K. pneumoniae* cells to the tested catheter specimens. The new nanobiosystems based on vanilla, patchouli and ylang-ylang essential oils could be of a great interest for the biomedical field, opening new directions for the design of film-coated surfaces with anti-adherence and anti-biofilm properties.

## 1. Introduction

The surface of the biomaterials implanted in the human body is rapidly covered by a proteinaceous conditioning film predisposing to microbial colonization that could lead to biofilm associated infections, produced by a wide range of microorganisms, including Gram-positive cocci and Gram negative bacilli. The biofilm associated infections are characterized by slow onset, middle intensity symptoms, chronic evolution and resistance to antibiotic treatment [1]. In order to overcome this challenging problem, there is an increasing need for the development of new antibiofilm compounds and strategies.

One of the promising approaches to combat biofilms related infections is the coating of medical devices surface with anti-biofilm pellicles releasing antimicrobial agents, able to reduce microbial adhesion and biofilm development.

Plant essential oils are complex, volatile, natural compounds formed by aromatic plants as secondary metabolites [2], largely used against infections produced by bacteria, fungi, and viruses [3,4,5,6,7,8,9]. The antimicrobial activity was attributed to four classes of active compounds represented by terpenes, terpenoids, phenylpropenes, and “others”, that have been shown to act by damaging the cell wall and membrane, inhibition of protein synthesis, or interference with intermediary metabolisms or DNA/RNA synthesis/function and to not exhibit selective pressure for the occurrence of microbial resistance [10,11].

Originating from Indonesia, Malaysia, Philippines, Seychelles Islands, Madagascar, India, Brazil, Japan and China, patchouli (*Pogostemon cablin* syn. *P. patchouli*, *P. heineanus*) essential oils contain almost exclusively bi- and tricyclic sesquiterpenoids, such as 10%–16% α-guaiene, β-caryophyllene, δ-cadinene, pogostol, and respectively 25%–46% (‒)-patchoulol, 6%–11% seychellene, α- and β-patchoulene, α- and β-patchoulene oxide, α-cedrene, (+)-*nor*-patchoulenol, patchoulenone, isopatchoulenone, patchoulone, and only traces of monoterpenoid hydrocarbons (limonene, α- and β-pinene) are found [12]. Pathchouli essential oil (PEO) is primarily used in cosmetics. Due to its main active sesquiterpenoid compounds, PEO exhibited *in vitro* antibacterial effects against a wide range of bacteria such as *Bacillus* sp., *Staphylococcus aureus*, *Streptococcus pyogenes*, *Proteus* sp., *Enterobacter aerogenes*, *Escherichia coli*, *Klebsiella pneumoniae*, *Pseudomonas aeruginosa*, *Salmonella typhi*, *Shigella dysenteriae* [13].

Oleoresin from vanilla (*Vanilla planifolia*) fruits is obtained by extraction with volatile organic solvents (benzene, petroleum ether, methylene chloride, *etc.*). Benzene extraction yield is approx. 6%. For use in perfumery, vanilla absolute (or “essential oil”) (VEO) is extracted from oleoresin with ethanol (60%–70% yield). VEO contains mainly aromatic derivatives such as vanillin (85%–87%), 4-hydroxybenzaldehyde (6%–9%), vanillic acid (4%–5%), 4-hydroxybenzyl methyl ether, ethylvanillin, piperonal, methyl anisate [14]. VEO highlighted an inhibitory effect against the quorum-sensing genes expression of the Tn-5 mutant of *Chromobacterium violaceum* CV026, a soil-borne Gram-negative bacterium [15]. Also, by disc-diffusion technique, VEO demonstrated *in vitro* antibacterial properties against *Enterobacter aerogenes*, *E. coli*, *Proteus vulgaris*, *P. aeruginosa*, *Streptococcus faecalis* [16].

GC-MS analysis of essential oil from the fresh flowers of ylang-ylang (*Cananga odorata* subsp. *genuina*) revealed a complex composition consisting mainly of aromatic derivatives (1%–13% benzyl acetate, 5%–10% *p*-cresyl methyl ether, benzoic acid, cinamyl acetate, methyl benzoate, benzyl benzoate, benzyl salicylate, eugenol, *trans*-anethole), monoterpenoids (1%–9% linalool, geranyl acetate, geraniol, 1,8-cineole), prenyl derivatives (isoprenyl acetate, prenyl acetate), and sesquiterpenoids (17%–22% germacrene D, 8%–24% α-farnesene, 5%–35% β-caryophyllene, 2%–10% α-humulene) [17]. *In vitro*, ylang-ylang essential oil inhibited biofilm formed by *E. coli* ATCC 25922, *S. aureus* ATCC 6538, *S. epidermidis* clinical isolated strain and *Candida albicans* ATCC10231 [18,19].

Recently, magnetic nanoparticles have been reported to be efficient carriers and delivery systems for antibiotics and essential oils [20,21]. The magnetic nanoparticles and antibiotics delivery nanosystems have been shown to improve the therapeutic index of antimicrobial drugs and to diminish their local and systemic side effects [22].

Therefore, the purpose of this study was to combine the unique properties of magnetic nanoparticles with the antimicrobial activity of three essential oils to obtain novel nanobiosystems that could be pelliculised on the surface of catheter pieces exhibiting an improved resistance to microbial adherence and biofilm development by *Staphylococcus aureus* and *Klebsiella pneumoniae* clinical strains.

## 2. Results and Discussion

Iron oxide nanostructures have attracted a lot of attention in the last decade due to their various applications in the medical field [23,24]. Recently, different applications for antimicrobial therapy have been developed, such as anti-biofilm surfaces or drug delivery systems [25,26,27,28,29,30,31,32,33,34,35,36,37]. In this context, nanobiosystems based on iron oxide nanoparticles and essential oils have been prepared in order to create novel anti-adherent surfaces.

**Figure 1:**
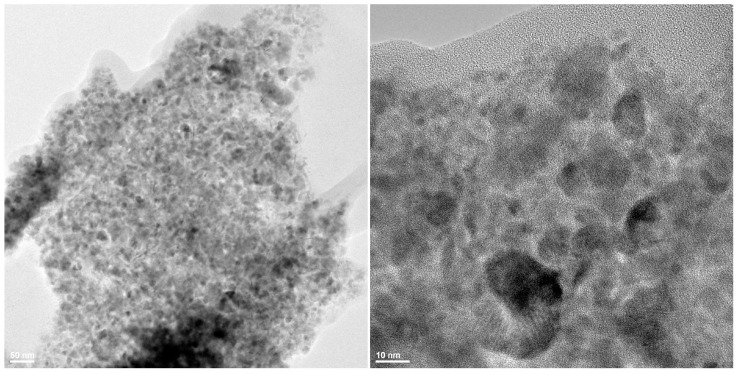
TEM images of Fe_3_O_4_@C_14_ nanostructures.

The obtained iron oxide nanoparticles functionalized with myristic acid (C_14_) have been characterized by TEM, XRD and TGA. TEM results are plotted in Figure 1. The images show that the prepared powder is in the nanometric range, the nanoparticles size is below 20 nm with a low tendency to create aggregates.

**Figure 2:**
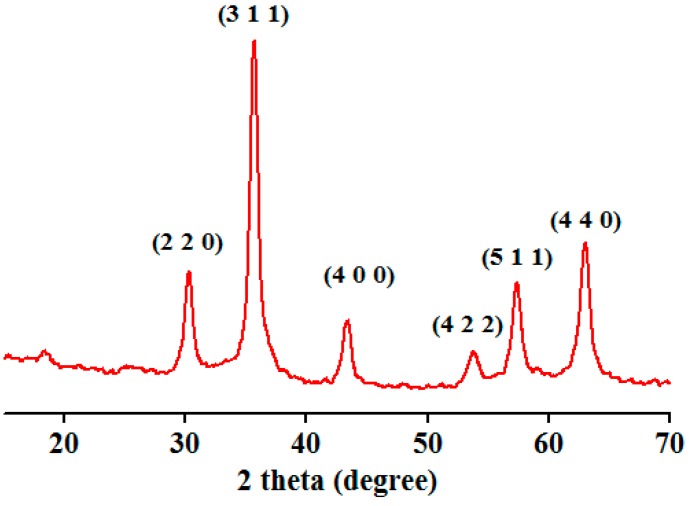
XRD pattern of Fe3O4@C14.

Figure 2 presents the XRD pattern of iron oxide nanostructures. Identified peaks can be assigned to magnetite nanoparticles [35,37]. From the XRD pattern it can be shown that the only crystalline phase presented in the prepared powder is magnetite.

**Figure 3:**
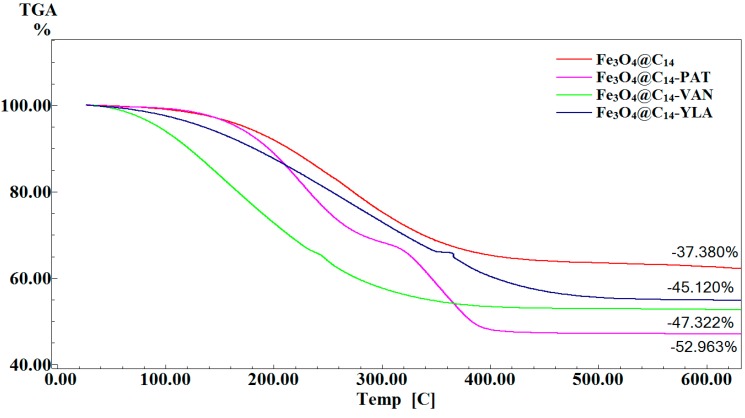
TG [C] analysis of Fe_3_O_4_@C_14_ and Fe_3_O_4_@C_14_-EOs.

TG analysis (Figure 3) was used in order to estimate the amount of EOs immobilized on the surface of Fe_3_O_4_@C_14_. According to this analysis the amount of PAT, VAN and YLA estimated at 600 °C was 7.74%, 9.94% and 15.583%. The percent of EOs immobilized on the surface of magnetite nanoparticles is strictly dependent on the polarity of EOs.

Despite their frequent presence as commensal bacteria on the human skin and mucous surfaces, staphylococci are also the most frequent causes of biofilm-associated infections, especially in intensive care unit patients [38]. *Klebsiella pneumoniae*, an important opportunistic pathogen, causes persistent infections associated to biofilms formed on in-dwelling medical devices [39]. Recent studies revealed that numerous plant derived compounds and essential oils exhibit increased antimicrobial properties [40,41,42,43,44,45,46,47,48]. They are relatively easy to obtain, have low mammalian toxicity, and degrade quickly in water and soil, making them relatively environmentally friendly [49]. The development of bacterial resistance toward natural plant products has been thus far documented only in a very limited number of cases (e.g., for reserpine).

Some essential oils have been found to be more effective against biofilm embedded bacteria, probably due to the fact that: (i) the extracellular matrix of the biofilm adsorbs the active components and increases their local concentration; (ii) the cell membrane or cell wall in biofilm cells is different from that in planktonic cells due to differential gene expression in the two cell types [11].

However, the therapeutic effects of the essential oils can be impaired by their high volatility, highlighting the necessity of novel vectoring stabilizing systems [50]. Recent studies have shown that nanoparticles can be used for the delivery of essential oils and for the enhancement of their activity at the site of infection, thus surpassing some of the main drawbacks for conventional antimicrobial agents, which are the development of multiple drug resistance and adverse side effects [51]. During this study we have assessed the antimicrobial activities of three essential oils stabilized by magnetic nanoparticles. Ylang ylang oil has been previously shown to exhibit anti-*S. aureus* and anti-*S. epidermidis* activity, in combination with lavender oil and clary sage oil [18], patchouli essential oils have been proved to be active against *Listeria monocytogenes*, *S. aureus* biofilms and exhibited repellent properties [18,52,53,54,55], while vanillin, the main flavour component of vanilla showed bacteriostatic activity against *Escherichia coli*, *Lactobacillus plantarum* and *Listeria innocua*, acting primarily by the dissipation of ion gradients and the inhibition of respiration [56].

**Figure 4:**
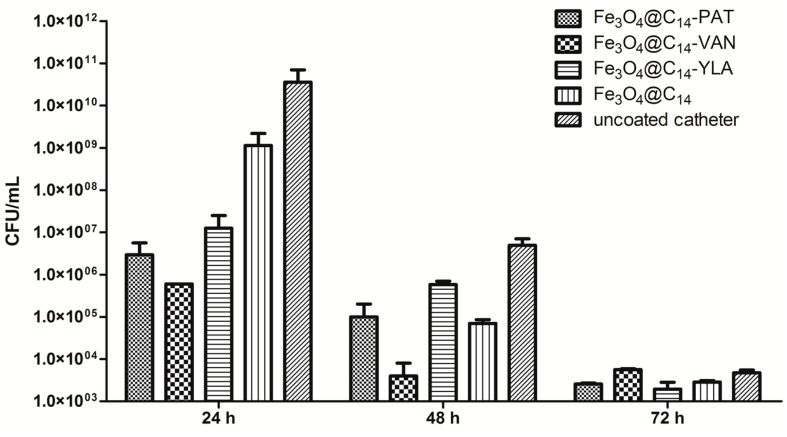
Number of *S. aureus* viable cells recovered from the biofilms developed on the catheter specimens coated with different essential oils containing nanosystems and on the uncoated catheters tested after 24, 48 and 72 h. Each column represents the average (Av) values of three replicates.

**Figure 5:**
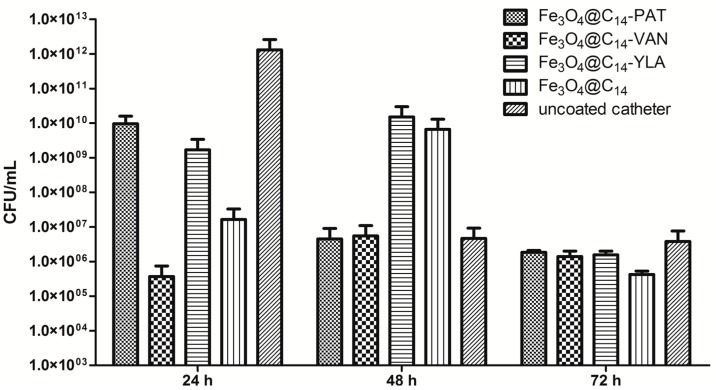
Number of *K. pneumoniae* viable cells recovered from the biofilms developed on the catheter specimens coated with different essential oils containing nanosystems and on the uncoated catheters tested after 24, 48 and 72 h. Each column represents the average (Av) values of three replicates.

In our study, the observed dynamics of *S. aureus* and *K. pneumoniae* biofilms presented a similar growth peak at 24 h, followed by a gradual decrease of bacterial density at 48 h and 72 h (Figure 4 and Figure 5).

However, it is to be noticed that the number of *K. pneumoniae* adhered cells quantified after 24 h has been >one log higher than in case of *S. aureus*.

The vanilla essential oil coated nanoparticles strongly inhibited both the initial adherence of *S. aureus* to the coated catheter surface (quantified at 24 h) and the development of the mature biofilm quantified at 48 h (Figure 4).

**Figure 6:**
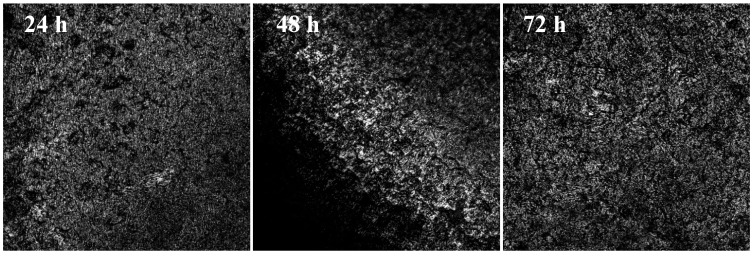
CLSM images of *S. aureus biofilm* developed on Fe_3_O_4_@C_14_-VAN coated substrata at 24 h, 48 h and 72 h; bacteria occur as punctiform, round cells distributed on the coated surface.

A direct correlation between the viable cell counts and CLSM results was observed. CLSM was used to obtain qualitative images on the distribution of the microbial biofilm on the coated catheteter surfaces *vs.* the uncoated ones. For example, in case of *S. aureus* biofilms developed on the catheter specimens coated with Fe_3_O_4_@C_14_-VAN*,* the CLSM images revealed that the biofilms examined at 24 h and 72 h were better represented than that developed at 48 h and more homogenously distributed on the coated surface (Figure 6).

The inhibitory effect of the vanilla essential oil loaded nanostructure can be interpreted as significant in accordance with US Pharmacopeia criteria for antimicrobial effectiveness [57], the number of the adherent cells being decreased by more than three/five logs at 24 h and by more than two/three logs at 48 h in comparison with the biofilm embedded cells developed on the catheter samples coated only with the magnetite nanostructures and respectively, on the uncoated catheter. Taking into account that the initial number of the bacterial cells added in the system was 1 × 10^8^–3 × 10^8^ CFU/mL, and the number of the attached bacteria is decreasing over time under the initial value we could state that the vanilla essential oil induced a bactericidal effect and a detachment of the bacterial cells from the biofilm.

The patchouli and ylang-ylang essential oils significantly inhibited the initial adherence phase of *S. aureus* biofilm development (Figure 4), the number of viable cells being decreased by at least two logs, in comparison with the biofilm developed on the catheter samples coated only with the magnetite nanostructures while at 48 h, the inhibitory effect was less evident, a biofilm inhibition of more than one log being noticed only in comparison with the uncoated catheter.

The tested oils did not exhibit any significant influence on the viability of bacterial cells embedded in biofilms after 72 h. These results demonstrate that the tested oils exhibit a bactericidal effect as long as they are released from the iron based nanocarrier which remains attached to the catheter surface. Thus, after the active compounds are consumed, the remaining viable cells are rebuilding the biofilm.

**Figure 7:**
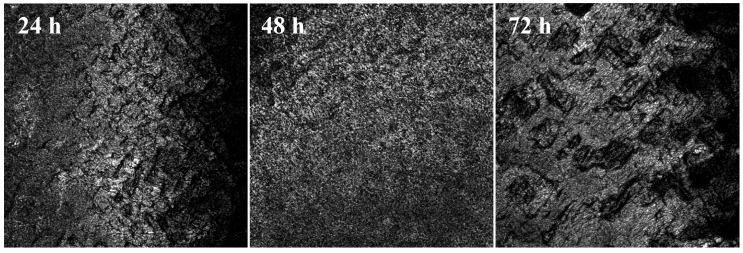
CLSM images of *K. pneumonia* biofilm developed on Fe_3_O_4_@C_14_-VAN coated substrata at 24 h, 48 h and 72 h; bacteria occur as punctiform, round cells distributed on the coated surface.

In case of *K. pneumoniae* biofilms, vanilla oil containing nanostructure proved to be the most potent inhibitor of the initial adherence of *K. pneumoniae* to the coated catheter specimens (the number of viable cells being decreased by five logs as compared to the uncoated control) (Figure 5), while patchouli and ylang-ylang oils exhibited a lower inhibition of the initial phase of biofilm development (the inhibitory effect being of two to three logs as compared to the uncoated control). However, similar to *S. aureus* biofilm, the effect seems to be more probably due to the bactericidal effect exhibited by the active compounds released from the coating rather than to the detachment of the adhered cells from the surface (Figure 7).

No significant inhibition of the mature biofilm development quantified at 48 h and 72 h has been observed in the presence of any of the three tested essential oils, the active compounds released from the pellicle being probably more rapidly consumed in their interaction with *K. pneumoniae* biofilm cells, more dense at 24 h (>10^12^ CFU/mL) as compared to *S. aureus* one (<10^10^ CFU/mL).

## 3. Experimental Section

### 3.1. Preparation of Iron Oxide@C_14_ Nanoparticles

Iron oxide@C_14_ nanoparticles were prepared according to our previously published papers [58]. Briefly, functionalized iron oxide nanoparticles were prepared by co-precipitation of Fe^+2^ and Fe^3+^ in basic aqueous dispersion of C_14_ (myristic acid).

### 3.2. Surface Modification of Catheter Pieces with Essential Oils Stabilized by Iron Oxide@C_14_ Nanoparticles

Surface modification of catheter pieces was performed according to our previously published papers [21,59]. Firstly, the 100 mg of iron oxide@C_14_ nanoparticles and 100 μL of essential oils (EOs) were dispersed in chloroform and mixed until complete evaporation of chloroform was achieved. This step was repeated for uniform loading of EOs on the surface of iron oxide@C_14_ nanoparticles. After 7 days prepared Fe_3_O_4_@C_14_-EOs were analyzed by TGA to estimate the amount of EOs immobilized on the surface of Fe_3_O_4_@C_14_. Secondly, after 7 days of drying at ambient temperature the layer of Fe_3_O_4_@C_14_-EOs on the catheter pieces was achieved by submerging the pieces in 10 mL of Fe_3_O_4_@C_14_-EOs fluid (Fe_3_O_4_@C_14_-EOs:CHCl_3_ = 1 mg/mL) and then the catheter pieces have been extemporaneously dried at room temperature. The rapid drying was facilitated by the convenient volatility of chloroform. The modified specimens were sterilized by ultraviolet irradiation for 20 min [51].

### 3.3. Characterization

#### 3.3.1. TEM

The transmission electron microscopy (TEM) images were obtained on finely powdered samples using a Tecnai^TM^ G2 F30 S-TWIN high resolution transmission electron microscope from FEI Company (Hillsboro, OR, USA). The microscope was operated in transmission mode at 300 kV with TEM point resolution of 2 Å and line resolution of 1 Å. The fine powder was dispersed into pure ethanol and ultrasonicated for 15 min. After that, diluted sample was put onto a holey carbon-coated copper grid and left to dry before TEM analysis.

#### 3.3.2. XRD

X-ray diffraction analysis was performed on a XRD 6000 diffractometer (Shimadzu, Kyoto, Japan) at room temperature. In all the cases, Cu Kα radiation (λ = 1.5406 Å at 15 mA and 30 kV) was used. The samples were scanned in the Bragg angle 2θ range of 10–80 degree.

#### 3.3.3. TGA

The thermogravimetric (TG) analysis of the Fe_3_O_4_@C_14_ and Fe_3_O_4_@C_14_-EOs was followed with a TG 449C STA Jupiter instrument (Netzsch, Selb, Germany). Samples were screened with 200 mesh prior to analysis, placed in an alumina crucible, and heated at 10 K·min^−1^ from room temperature to 800 °C, under the flow of 20 mL·min^−1^ of dried synthetic air (80% N_2_ and 20% O_2_).

### 3.4. Microbial Biofilms Assay

The microbial adherence ability and biofilm development on the functionalized surfaces have been investigated by a culture-based method, using Gram positive (*Staphylococcus aureus* 246) and Gram-negative (*Klebsiella pneumoniae* 11) bacterial clinical strains. These strains have been identified using the automatic VITEK II identification system. The specimens of equal area have been distributed in the multi-well plastic plates, and submitted to UV sterilization for 20 min. Thereafter, the liquid culture medium (nutrient broth) was added over the slide specimens. Two wells were inoculated with a microbial inoculum with a density corresponding to 0.5 MacFarland density (1 × 10^8^–3 × 10^8^ CFU/mL) prepared in sterile saline. The obtained microbial strain: culture medium: specimen system was incubated at 37 °C for 24 h, in order to allow microbial strains to multiply and adhere to the samples distributed in each well. The colonized specimen was washed in order to remove the nonadherent bacteria, moved in fresh culture medium and further incubated at 37 °C. After 24 h, 48 h and 72 h, respectively, the specimens were extracted from the culture medium, washed three times in sterile saline, in order to remove the non-adherent bacteria. Ten-fold dilutions were performed from the cultures recovered after the multiplication of microbial cells adhered to the tested substrata in order to count the Colony Forming Units (CFU) and to assess the viable cell counts (VCCs) of the respective cultures. To this purpose, the adhered cells have been removed from samples by vortexing and brief sonication. Serial dilutions ranging from 10^−1^ to 10^−30^ of the obtained inocula have been spotted on Muller-Hinton agar, incubated for 24 h at 37 °C and assessed for VCCs. An amount of 5 µL of the chosen dilution was spotted in triplicates on the solid medium. The resulting colonies have been numbered and the average value was submitted to dilution and volume correction. The final value was expressed in CFU/mL. We could assess this way the influence of different tested substrata on the adherence and the dynamics of microbial biofilm development by the selected microbial strains. All experiments were performed on different occasions using three biological replicates. The qualitative examination of biofilm development on the coated catheter surfaces was assessed by CLSM. For our experiments we have used a TCS SP confocal laser scanning microscope (Leica, Wetzlar, Germany) equipped with a He-Ne laser (633 nm) and a PL FLUOTAR40X, NA 0.75 objective. In order to evaluate the biofilm development on the coated *vs.* control, uncoated catheter surfaces, we have acquired reflection images from each sample surface. No staining of the bacteria was used.

## 4. Conclusions

The catheter colonization capacity of *S. aureus* and *K. pneumoniae* strains, particularly in the initial adherence phase (in case of all three essential oils), but also the development of the mature biofilm (in case of vanilla essential oil) were strongly inhibited by the essential oil-containing nanostructures, suggesting the potential of the obtained nanophytosystems for the design of coatings resistant to microbial colonization with different applications in medicine, food and pharmaceutical industry.
